# A New [PMo_12_O_40_]^3−^-Based Ni^II^ Compound: Electrochemical and Photocatalytic Properties for Water Pollutant Removal

**DOI:** 10.3390/molecules30102172

**Published:** 2025-05-15

**Authors:** Guoqing Lin, Shufeng Liu, Dai Shi, Ying Yang, Fangle Yu, Tong Lu, Xiao-Yang Yu, Yuguang Zhao

**Affiliations:** 1 Department of Materials Science and Engineering, Jilin University, Changchun 130025, China; linguoqing@jlict.edu.cn (G.L.); shidai@jlict.edu.cn (D.S.); 2 Jilin Institute of Chemical Technology, Jilin 132022, China; yangying@jlict.edu.cn (Y.Y.); yufengli123@126.com (F.Y.); 3 Jilin Provincial Institute of Water Resources and Hydropower Investigation, Changchun 132000, China; liushufeng002@163.com; 4 State Key Laboratory of Supramolecular Structure and Materials, Jilin University, Changchun 130012, China; lutong@jlu.edu.cn

**Keywords:** wastewater treatment, Keggin-type polyoxometalate, Hirshfeld surface analysis, electrocatalysis, amperometric sensor, photocatalysis

## Abstract

A polyoxometalate-based metal–organic complex with the ability to treat pollutants in water was obtained under hydrothermal conditions, namely [Ni(H_2_L)(HL)_2_](PMo_12_O_40_)·3H_3_O·4H_2_O (**1**) (H_2_L = 4,4′-(1H,1′H-[2,2′-biimidazole]-1,1′-diyl)dibenzoicacid). Structural analysis reveals that the [Ni(H_2_L)(HL)_2_] units are interconnected into a 2D layer via hydrogen bonds between adjacent carboxyl groups and water molecules of crystallization. [PMo_12_O_40_]^3−^ anions are embedded within the larger pores of the layer and are connected to the adjacent layers through hydrogen bonds, ultimately expanding the structure into a 3D supramolecular architecture. The intermolecular interactions were studied via Hirshfeld surface (HS) analysis. Electrochemical performance tests reveal that **1** exhibits electrocatalytic activity toward the oxidation and reduction of diverse pollutants in water, including NO_2_^−^, Cr(VI), BrO_3_^−^, Fe(III), and ascorbic acid (AA). Additionally, it can also serve as an amperometric sensor for the detection of BrO_3_^−^ and Cr(VI). Photocatalytic studies reveal that compound **1** functions as a bifunctional photocatalyst, which not only achieves efficient degradation of organic dyes but also demonstrates remarkable reduction efficiency for toxic Cr(VI). Compound **1** demonstrates significant potential for practical water remediation applications.

## 1. Introduction

Water is an essential resource for human survival and development on Earth [1,2,3]. However, with the rapid development of industrialization and urbanization, the chemical pollution in the water environment has become increasingly severe, posing a significant threat to both aquatic organisms and human health [4,5,6]. Chemical pollutants in water primarily consist of inorganic pollutants (e.g., heavy metal ions, inorganic anions) and organic pollutants (e.g., organic dyes, pharmaceutical intermediates) [7,8,9,10]. Given that these pollutants are resistant to natural degradation and removal, and exhibit persistence and bioaccumulation, the development of efficient multifunctional materials for detecting and removing water pollutants is crucial for preventing related diseases and ensuring environmental safety [11,12,13].

Polyoxometalates (POMs) have attracted significant attention in materials science owing to their structural versatility and excellent redox properties, and are widely used in diverse fields, including electrochemistry, catalysis, optics, magnetism, biomedicine, and so on [14,15,16,17]. POM-based systems exhibit great potential for water environment restoration due to their ability to efficiently remove organic and inorganic pollutants via photocatalysis and electrocatalysis. Upon light absorption (photocatalysis) or electrical energy input (electrocatalysis), these systems generate electron-hole pairs (e^−^/h^+^), which subsequently produce highly oxidizing reactive radicals, including hydroxyl radicals (·OH) and superoxide radicals (O_2_^−^), enabling effective pollutant removal [18]. The construction of POM-based metal–organic complexes (POMOCs) through the combination of POMs and metal–organic complexes (MOCs) can create additional active sites via synergistic assembly between the MOC units and POM anions [19,20]. On the one hand, metal–organic coordination units can optimize the photocatalytic activity of hybrid materials by regulating the energy gap between the highest occupied molecular orbital (HOMO) and the lowest unoccupied molecular orbital (LUMO) of POMs [6,21,22,23]. On the other hand, the synergistic effect between POMs and MOCs, such as electron transfer and interfacial interaction, can significantly enhance the electrocatalytic performance of materials [24,25]. Moreover, this approach effectively reduces the solubility of unmodified POMs in polar solvents, thereby significantly enhancing the feasibility of their practical applications [26]. By rationally designing the structure and composition of POMOCs, it is possible to develop highly efficient and stable novel catalysts for the removal of water pollutants.

The selection of functional organic ligands is crucial for the formation and application of POMOCs. Heterocyclic aromatic carboxylic acid ligands, with their electronic effects and extended π-conjugated systems, can significantly enhance the performance of POMOCs, improve their performance in applications such as photocatalysis, and modulate the redox potentials of POM units. Moreover, the excellent coordination ability of these ligands, along with the π···π stacking interactions of their aromatic rings and the hydrogen-bonding interactions of their carboxyl groups, collectively stabilize the structure of POMOCs and enhance their stability [11,27,28,29,30].

Therefore, the semi-rigid heterocyclic aromatic carboxylic acid ligand, 4,4′-(1H,1′H-[2,2′-biimidazole]-1,1′-diyl)dibenzoic acid (H_2_L), was employed, and a multifunctional Keggin-based POMOC, namely [Ni(H_2_L)(HL)_2_](PMo_12_O_40_)·3H_3_O·4H_2_O (**1**), was successfully obtained under hydrothermal conditions. The structure was characterized, and the surface weak interactions were analyzed utilizing Hirshfeld surface software (CrystalExplorer 21.0 program). The electrocatalytic activity, amperometric sensing performance, and photocatalytic properties of compound **1** were investigated. The results show that compound **1** can not only effectively remove inorganic and organic pollutants in water through electrocatalysis and photocatalysis, but can also detect Cr(VI) and bromate ions in water with high sensitivity via amperometric sensing, showing multifunctional POMOC material properties with great potential in repairing water environment.

## 2. Results and Discussion

### 2.1. Crystal Structure of Compound **1**

**Figure 1:**
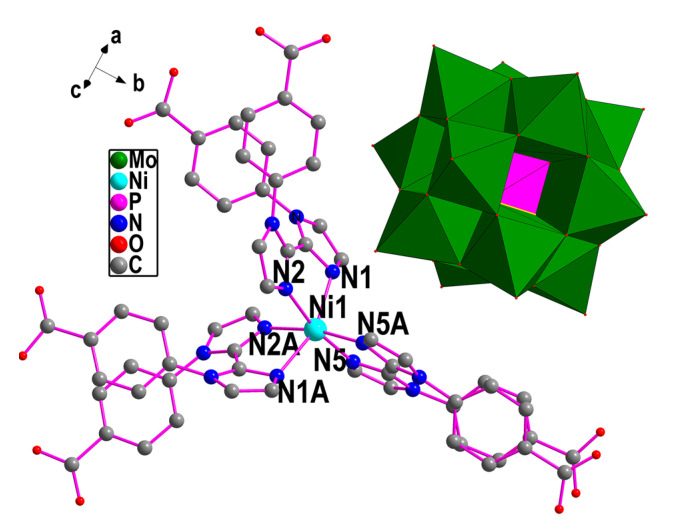
The structure of compound **1** (hydrogen atoms and water molecules are omitted for clarity, symmetric code: A, 1 − *x*, *y*, 0.5 − *z*).

Single-crystal X-ray diffraction reveals that compound **1** crystallizes in the monoclinic C2/c space group. The asymmetric unit is composed of a half Ni^2+^, a half Keggin-type polyoxometalate anion [PMo_12_O_40_]^3−^ (simplified as PMo_12_), one HL^−^, a half H_2_L, and two and three halves lattice water molecules. One-and-a-half water molecules are protonated for charge balance (Figure 1).

In PMo_12_, the central P atom adopts a tetrahedral coordination geometry surrounded by eight oxygen atoms, with each site exhibiting an occupancy factor of 0.5. The bond lengths of P-O and Mo-O are all within the normal ranges [31]. The oxidation states of Mo and P are +VI and +V, respectively, as determined by bond valence sum calculations [32].

**Figure 2:**
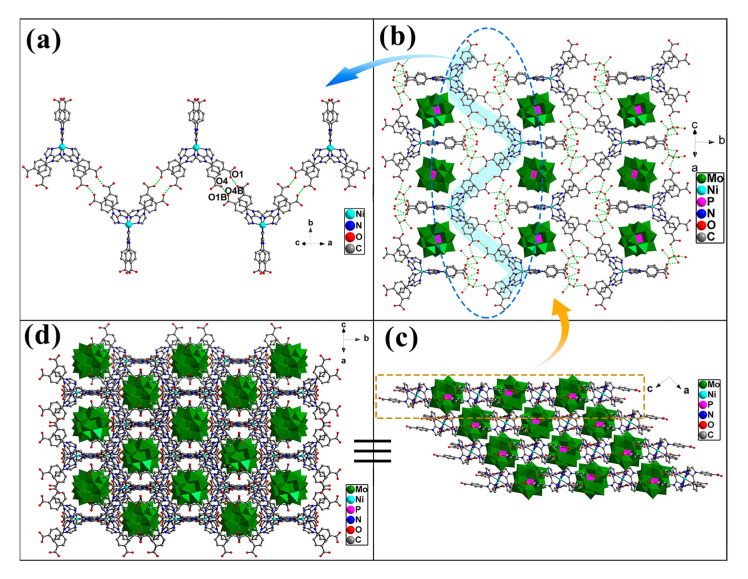
(**a**) A 1D wavelike supramolecular chain formed by [Ni(H_2_L)(HL)_2_] units. (**b**) Hydrogen bonds connect the 1D supramolecular chains into 2D porous supramolecular layers, within which the PMo_12_ are embedded (symmetric code: B, 1.5 − *x*, 0.5 − *y*, −*z*). (**c**,**d**) 3D supramolecular structure of **1**.

In compound **1**, Ni^2+^ is six coordinated by six N atoms (N1, N1A, N2, N2A, N5, and N5A, symmetric code: A, 1 − *x*, *y*, 0.5 − *z*) from three organic ligands to form an octahedral geometry. The Ni-N distances in the range of 2.045(6)–2.183(6) Å are comparable to those reported in the literature [28,33]. Three organic ligands adopt chelating coordination modes, utilizing six imidazole-N atoms to chelate one Ni^2+^ ion and form a [Ni(H_2_L)(HL)_2_] unit. All carboxyl groups in the ligands remain uncoordinated, leading to a discontinuous structural arrangement between the Ni^2+^ ions and the organic ligands. Neighboring [Ni(H_2_L)(HL)_2_] units are connected into a 1D wavelike chain by the hydrogen bonds between the adjacent carboxyl groups, respectively, as shown in Figure 2a. Hydrogen bonds formed between the carboxyl groups and the interstitial crystallization water molecules act as adhesive agents to connect adjacent 1D supramolecular chains into a 2D supramolecular layer structure with two types of pores, as shown in Figure S1 and Figure 2b, where PMo_12_ are filled in the larger pores and hydrogen bond to the layers. Compound **1** is self-assembled into a 3D supramolecular structure via intermolecular hydrogen bond interactions (Figure 2c,d).

### 2.2. Hirshfeld Surface Analysis

The Hirshfeld surface (HS) analysis was performed using CrystalExplorer 21.0 software to elucidate the intermolecular interaction forces within the supramolecular structure of compound **1**. This included calculating the HS and 2D fingerprint (FP) results of the compound, visualizing the intermolecular interactions, and distinguishing the type of interaction and its percentage contribution form [34].

**Figure 3:**
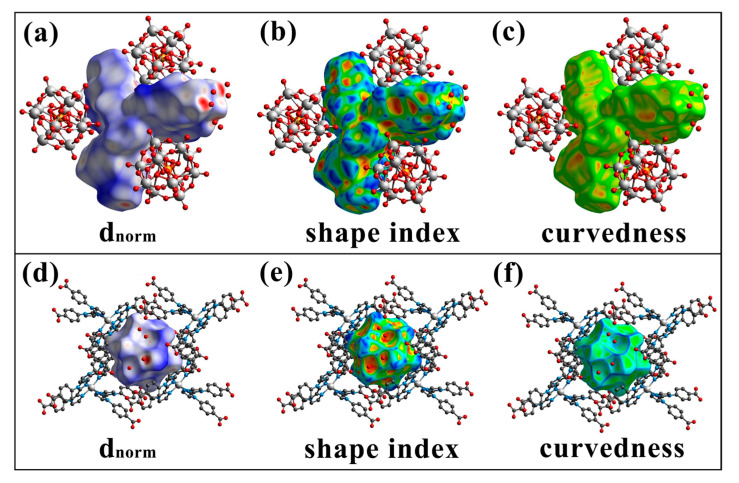
Hirshfeld surface maps (**a**,**d**), shape index maps (**b**,**e**), and curvedness maps (**c**,**f**) for the [Ni(H_2_L)(HL)_2_] unit and the PMo_12_ unit in crystal structure **1**.

The surfaces of [Ni(H_2_L)(HL)_2_] unit and PMo_12_ are calculated, respectively. As shown in Figure 3, the intermolecular interactions of the HS for the [Ni(H_2_L)(HL)_2_] unit and PMo_12_ were visualized through *dnorm* (the normalized contact distance), shape index, and curvedness analyses. *Dnorm* is calculated based on two types of distances: internal (*di*) and external (*de*). In the *dnorm* map, the red, white, and blue regions correspond to distances that are less than, equal to, or greater than the sum of the van der Waals radii of the relevant atoms, respectively [35,36]. The shape index is used to characterize intermolecular π···π interactions, which are depicted as symmetrical red and blue triangles on the shape index map. The curvature map visualizes the shape index, highlighting planar regions within the molecule as distinct features. HS analysis of the crystal structure reveals that the intermolecular interactions on the two surfaces are predominantly hydrogen bonds. Since the surface of the polyoxometalate anion is primarily composed of oxygen atoms, the surface interactions are mainly characterized by oxygen-centered hydrogen bonds or other non-covalent forces.

**Figure 4:**
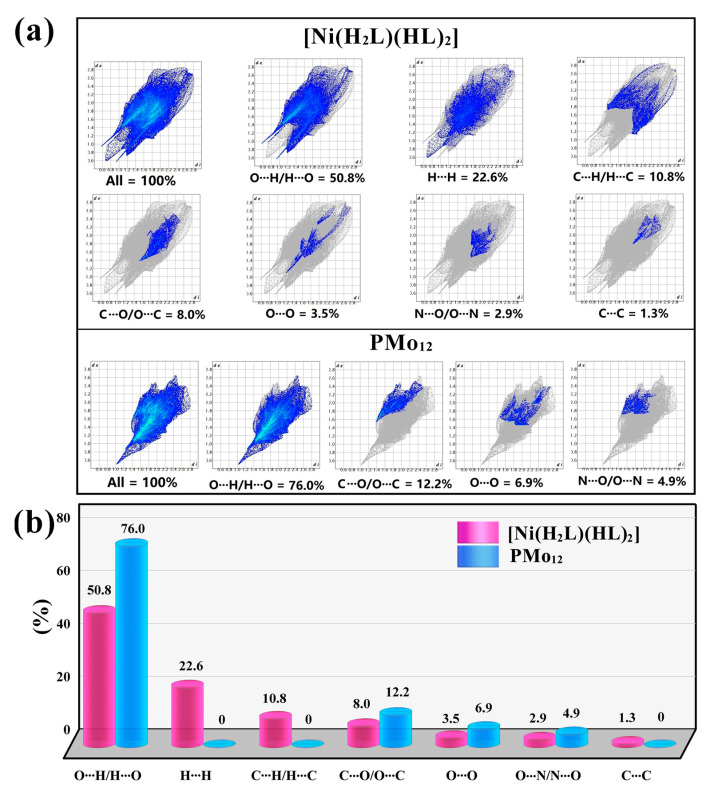
The 2D fingerprint plots (**a**) and histograms (**b**) depicting the contribution ratios of supramolecular interactions in the [Ni(H_2_L)(HL)_2_] unit and the PMo_12_ unit.

As shown in Figure 4, the 2D fingerprint plot reveals that the [Ni(H_2_L)(HL)_2_] unit is primarily characterized by O···H/H···O (50.8%), H···H (22.6%), C···H/H···C (10.8%), and C···O/O···C (10.8%) interactions, with fewer additional contacts. In contrast, PMo_12_ exhibits only four intermolecular interactions: O···H/H···O (76.0%), C···O/O···C (12.2%), O···O (6.9%), and N···O/O···N (4.9%), with no other forces present.

### 2.3. Powder X-Ray Diffraction and FT-IR Spectra

The measured PXRD pattern of compound **1** exhibits excellent agreement with the simulated pattern derived from single-crystal data, indicating high phase purity of the compound (Figure S2) [37].

The FT-IR spectra of **1** are shown in Figure S3. The broad peaks around 3443 cm^−1^ can be attributed to the *ν*(O-H) stretching vibration of the free water molecules in **1** [38]. The bands in the range of 1709–1422 cm^−1^ can be assigned to the carboxyl groups of the organic ligands. Specifically, the peak at 1709 cm^−1^ is attributed to the C=O stretching vibration of the partially deprotonated carboxyl groups [39,40]. The peaks at 1512 cm^−1^ and 1422 cm^−1^ correspond to the characteristic stretching vibration peaks of *νas*(COO-) (asymmetric stretching) and *νs*(COO-) (symmetric stretching) of the carboxylate groups, respectively [41]. The peaks observed near 1273 cm^−1^ and 1173 cm^−1^ can be assigned to the C-N stretching vibration of the imidazole ring and the in-plane bending vibration of the aromatic ring, respectively [40,41,42]. The characteristic bands at 1058, 960, 884, and 804 cm^−1^ can be assigned to *vas*(P-O), *vas*(Mo=Od), *vas*(Mo-Oc-Mo), and *vas*(Mo-Ob-Mo), respectively [43,44].

### 2.4. TG Analyses

Thermogravimetric (TG) analysis of compound **1** was performed at a heating rate of 10 °C min^−1^ from room temperature to 700 °C in air. As shown in Figure S4, there are two steps in weight loss processes. The first weight loss step corresponds to the loss of the crystallization water molecules (from 25 °C to 185 °C, obsd., 4.03%, calcd., 4.12%). The second weight loss step is attributed to the decomposition of the organic ligands (from 292 °C to 598 °C, obsd., 36.21%, calcd., 35.74%). In the process of TG analysis, differential scanning calorimetry (DSC) was used to monitor the heat changes during the process. The results show that there are three obvious thermal changes, which can be attributed to the loss of water molecules (132 °C) and the cleavage of chemical bonds in organic ligands (432 °C and 522 °C) [45].

### 2.5. Electrochemical Properties

#### 2.5.1. Cyclic Voltammetric Behaviors

**Figure 5:**
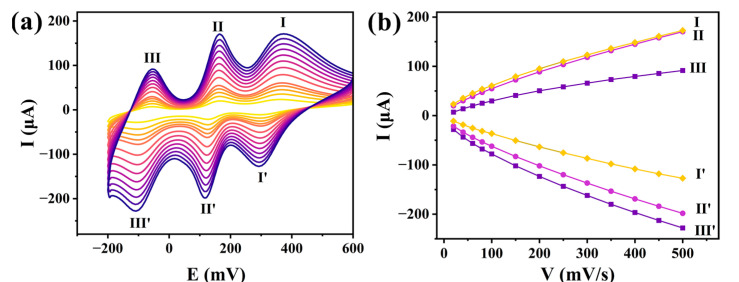
(**a**) The CV of **1**-CPE in a 0.1 M H_2_SO_4_ + 0.5 M Na_2_SO_4_ electrolyte solution at the different scan rates (from inner to outer: 20, 40, 60, 80, 100, 150, 200, 250, 300, 350, 400, 450, and 500 mV·s^−1^). (**b**) The plots of the anodic and cathodic peak currents vs. scan rates.

The electrochemical properties of **1**-CPE were evaluated in a 0.1 M H_2_SO_4_ + 0.5 M Na_2_SO_4_ aqueous solution. As shown in Figure 5a, the cyclic voltammograms of **1**-CPE measured at scan rates ranging from 20 to 500 mV s^−1^ reveal three distinct redox couples (I-I’, II-II’, and III-III’) with half-wave potentials (E1/2 = (Epa + Epc)/2, at 200 mV·s^−1^) of +335, +142, and −79 mV, respectively. The electrochemical responses correspond to two single-electron transfer processes and one two-electron transfer process, as evidenced by the peak current ratios and potential separations [46]. The three processes can be expressed as follows:

PMo_12_^VI^O_40_^3−^ + H^+^ + e^−^ → HPMo_11_^VI^Mo^V^O_40_^3−^ (1)

HPMo_11_^VI^Mo^V^O_40_^3−^ + 2H^+^ + 2e^−^ → H_3_PMo_9_^VI^Mo_3_^V^O_40_^3−^ (2)

H_3_PMo_9_^VI^Mo_3_^V^O_40_^3−^ + H^+^ + e^−^ → H_4_PMo_8_^VI^Mo_4_^V^O_40_^3−^ (3)

As the scan rate was increased from 20 to 500 mV·s^−1^, a systematic shift in peak potentials was observed, with the cathodic peak moving toward more negative potentials and the anodic peak shifting toward more positive potentials. The CV profiles maintained their characteristic shape without significant distortion, indicating stable electrochemical behavior over the investigated scan rate range. As shown in Figure 5b, there is a linear dependence of the peak currents (Ip) for all three redox couples on the scan rate, which is characteristic of a surface-controlled process [28].

#### 2.5.2. Electrocatalytic Properties

**Figure 6:**
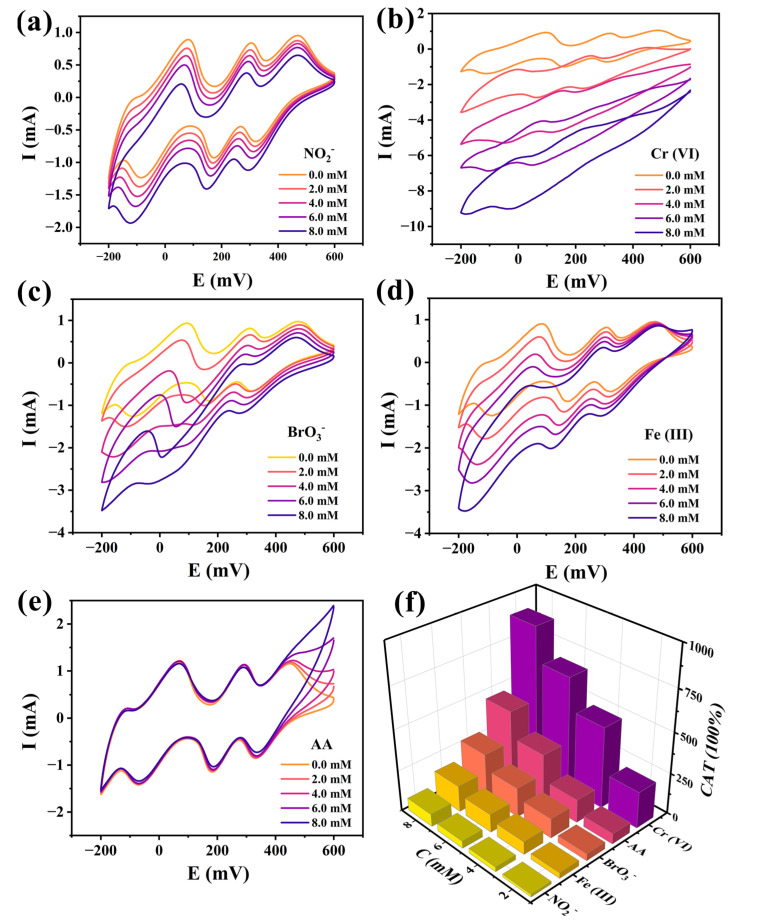
The CVs of **1**-CPE in 0.1 M H_2_SO_4_ + 0.5 M Na_2_SO_4_ electrolyte solutions containing NO_2_^−^ (**a**), Cr(VI) (**b**), BrO_3_^−^ (**c**), Fe(III) (**d**), and AA (**e**) in the concentration range from 0 to 8.0 mM (identical scanning rate: 200 mV·s^−1^). (**f**) Histogram of the CATs of **1**-CPE.

The electrocatalytic performances of **1**-CPE were also investigated at a scan rate of 200 mV·s^−1^. As seen in Figure 6a–d, with the increase in the concentrations of NO_2_^−^, Cr(VI), and BrO_3_^−^, the reduction peak currents for the I-I’, II-II’, and III-III’ peaks progressively increased, while the corresponding oxidation peak currents gradually decreased. The result indicated that all three redox states of PMo_12_ actively participate in the electrocatalytic reduction of NO_2_^−^, Cr(VI), and BrO_3_^−^. With the increase in the concentration of Fe(III), only the reduction peak currents for the II-II’ and III-III’ peaks increased significantly, while the reduction peak currents for the I-I’ peaks remained almost unchanged. This suggests that only the second and third redox states of PMo_12_ participate in the electrocatalytic reduction of Fe(III). As shown in Figure 6e, the oxidation peak current of **1**-CPE gradually increased with the addition of AA, demonstrating its effective electrocatalytic oxidation capability. The results collectively prove that **1**-CPE possesses distinctive bifunctional electrocatalytic properties, enabling efficient reduction and oxidation reactions.

The electrocatalytic efficiencies (CATs) of **1**-CPE towards NO_2_^−^, Cr(VI), BrO_3_^−^, Fe(III), and AA were evaluated using the following formula: CAT = 100% × [Ip(C, substrate)-Ip(C)]/Ip(C), where Ip(C, substrate) and Ip(C) represent the anodic peak current intensities in the presence and absence of the substrate (NO_2_^−^, BrO_3_^−^, Cr(VI), Fe(III), or AA), respectively. As shown in Figure 6f, with the concentrations of the substrate increasing from 2.0 to 8.0 mM, the CATs (%) for **1**-CPE were estimated to be 15.51, 28.35, 44.53, and 76.90 toward NO_2_^−^; 33.59, 78.71, 111.15, and 160.19 toward Fe(III); 42.59, 120.31, 181.00, and 272.00 toward BrO_3_^−^; 64.64, 142.15, 301.64, and 459.95 toward AA; and 224.02, 498.51, 690.44, and 895.64 toward Cr(VI), respectively. This systematic increase in catalytic efficiency across all analytes demonstrates the concentration-dependent behavior of **1**-CPE’s electrocatalytic performance.

#### 2.5.3. Electrochemical Sensing Activities

**Figure 7:**
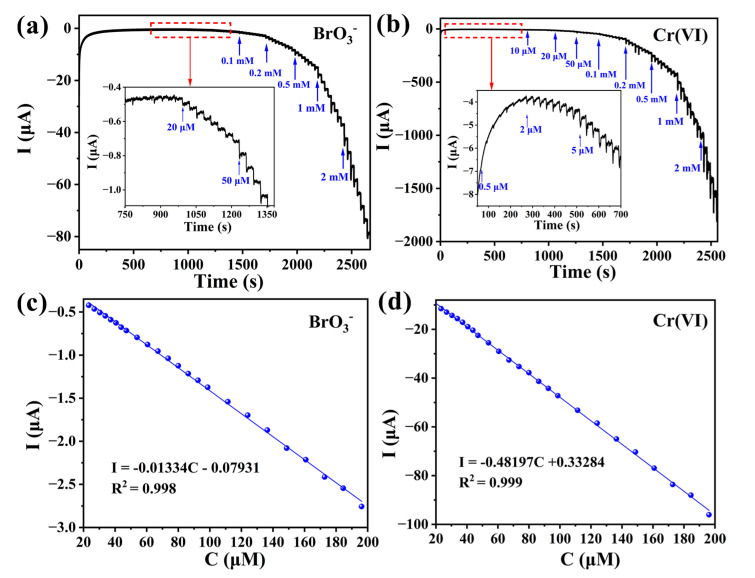
Current responses upon continuous addition of BrO_3_^−^ (**a**) and Cr(VI) (**b**) in the electrolyte solution, and correlation diagrams between current and concentration of BrO_3_^−^ (**c**) and Cr(VI) (**d**) detected using **1**-CPE.

The current sensing performances of **1**-CPE were investigated based on the electrocatalytic results, which showed that **1**-CPE exhibited an obvious steady-state current response to BrO_3_^−^ and Cr(VI), as shown in Figure 7a,b. The experiments were conducted in a continuously stirred electrolyte solution, where BrO_3_^−^ and Cr(VI) were added at 30 s intervals under the optimal potential of −0.05 V.

With the continuous addition of BrO_3_^−^ and Cr(VI), the current response value gradually increases and quickly reaches the steady-state current. As shown in Figure 7c,d, the relationships between the current response and the concentrations of BrO_3_^−^ (20–200 µM) and Cr(VI) (20–200 µM) are linear within the corresponding concentration ranges. The limits of detection (LODs) are 2.35 μM for BrO_3_^−^ (I(µA) = −0.01334C (µM) − 0.07931 (R^2^ = 0.998)) and 1.17 µM for Cr(VI) (I(µA) = −0.48197C (µM) + 0.33284 (R^2^ = 0.999)). The LODs were calculated using the following Equation (4):

(4) $$LOD=\frac{S/N\cdot \sigma }{k}$$

S/N represents the signal-to-noise ratio (with the value of 3), *σ* is the standard deviation, and *k* represents the linear regression slope.

**Figure 8:**
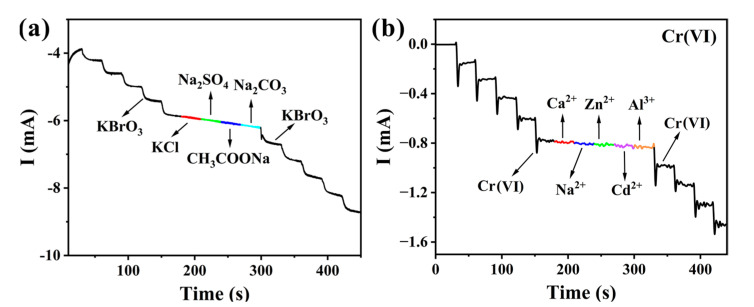
Current responses of **1**-CPE for BrO_3_^−^ (**a**) and Cr(VI) (**b**) upon the addition of potential interference substances into 0.1 M H_2_SO_4_ + 0.5 M Na_2_SO_4_ electrolyte solutions.

The selectivity and anti-interference capabilities of **1**-CPE as an electrochemical sensor for BrO_3_^−^ and Cr(VI) were systematically investigated. The current response exhibited a distinct and gradual decrease upon the addition of BrO_3_^−^ to the electrolyte solution. After 180 s, when KCl, Na_2_SO_4_, CH_3_COONa, and Na_2_CO_3_ were sequentially introduced into the solution at regular intervals, no significant changes in the current response were observed upon the addition of these interfering species (Figure 8a). Furthermore, the current response continued to decrease significantly with the subsequent addition of BrO_3_^−^ ions. In addition, Ca^2+^, Na^2+^, Zn^2+^, Cd^2+^, and Al^3+^ were introduced as interfering ions to evaluate the anti-interference capability of the current sensor for Cr(VI) detection. When Cr(VI) was added to the electrolyte solution, the current response decreased significantly. However, the introduction of the interfering ions resulted in negligible changes in the current response (Figure 8b). These results indicate that the **1**-CPE can be used as an electrochemical sensor with good selectivity and strong anti-interference for the determination of BrO_3_^−^ and Cr(VI).

### 2.6. Photocatalytic Performance

#### 2.6.1. Optical Band Gap

The semiconducting properties of compound **1** were investigated by determining its band gap energy (Eg) using diffuse reflectance spectroscopy. In this method, Eg was calculated from the intersection point between the energy axis and the linear extrapolation of the absorption edge in the plot of the Kubelka–Munk function (F) versus photon energy (E). As shown in Figure S5, the calculated Eg is 2.44 eV, indicating that compound **1** exhibits semiconductor behavior and has potential as a photocatalyst [30].

#### 2.6.2. Photocatalytic Degradation of MB

**Figure 9:**
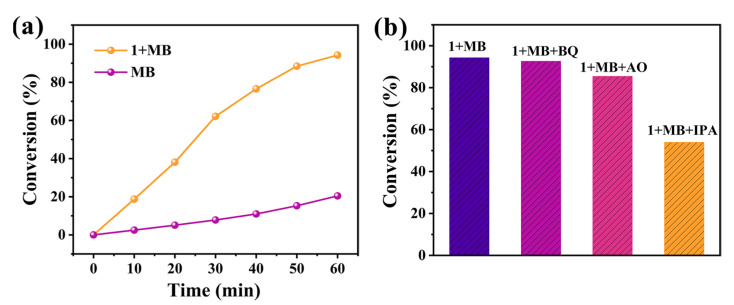
(**a**) The photocatalytic degradation rate of MB solution (40 ppm) with and without photocatalyst. (**b**) Photodegradation of the MB with different scavengers by using **1** as the photocatalyst.

The photocatalytic degradation capability of compound **1** towards organic dye pollutants was investigated by using methylene blue (MB) as the target organic dye. As shown in Figure S6, 5.0 mg of compound **1** was added to a MB solution (40 ppm) and left undisturbed in the dark for 12 h to ensure that the system reached adsorption–desorption equilibrium (the concentration decreased by 28.96%). Subsequently, the photocatalytic experiment was initiated. After 60 min, the photocatalytic degradation rate of the MB solution reached approximately 94.25% (Figure 9a). In contrast, the degradation rate of the MB solution was only approximately 20.43% after 60 min in the absence of any catalyst. The experimental results demonstrate that compound **1** exhibits significant photocatalytic degradation activity towards MB.

The photocatalytic degradation experiments of compound **1** were also conducted on MB solutions with lower concentrations (5 ppm, 10 ppm, and 20 ppm). As shown in Figure S6, when the MB concentration was 5 ppm or 10 ppm, compound **1** achieved nearly complete adsorption in the dark within 12 h. However, at a higher concentration of MB (20 ppm), adsorption–desorption equilibrium was reached in the dark within 12 h, with a concentration decrease of 59.39%. Subsequent photocatalytic degradation under light irradiation enabled 93.51% MB removal within 60 min. These results demonstrate that compound **1** can achieve nearly complete adsorption of MB in solutions with concentrations less than 10 ppm. When the concentration exceeds 20 ppm, the synergistic effect of adsorption and photocatalytic degradation is essential for efficient MB removal.

PXRD and FTIR characterization of the recovered compound **1** after the photocatalytic reaction revealed no changes in its characteristic peaks (see Figure S7), confirming its structural stability as a photocatalyst.

To further investigate the primary reactive species in the photocatalytic degradation of MB by compound **1**, benzoquinone (BQ, 15 mg), ammonium oxalate (AO, 15 mg), and isopropanol (IPA, 2.0 mL) were respectively introduced into the reaction system at the beginning of the photocatalytic experiments to scavenge superoxide radicals (·O_2_^−^), photogenerated holes (h^+^), and hydroxyl radicals (·OH) [30]. The experimental results revealed that the degradation rate of MB was most significantly inhibited by IPA, whereas BQ and AO exhibited only minor effects (Figure 9b). These findings indicate that ·OH plays a dominant role in the photocatalytic degradation process compared with ·O_2_^−^ and h^+^, suggesting that ·OH is the primary active species.

In order to evaluate the reusability of the catalyst, the catalyst was washed with NaCl solution and DMF to remove residual organic matter after each experiment and then dried and reused. As shown in Figure S8, after five cycles, the degradation efficiency of MB only decreased slightly to 88.25%, indicating that compound **1** has excellent cycle stability and photocatalytic activity.

#### 2.6.3. Photocatalytic Reduction of Cr(VI)

**Figure 10:**
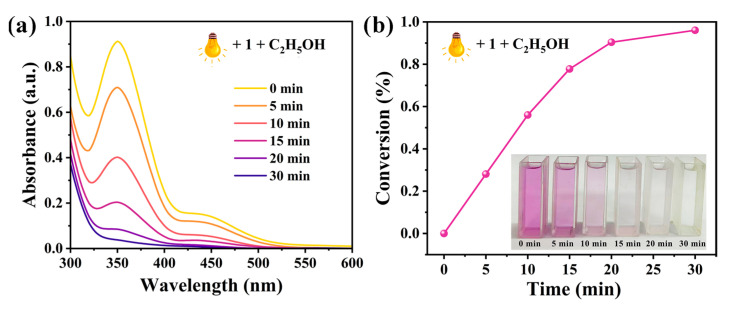
(**a**) UV-Vis absorption spectra of Cr(VI) solution mixed with **1** and C_2_H_5_OH under the irradiation of 300 W xenon lamp. (**b**) The corresponding photocatalytic degradation efficiency and the color changes in Cr(VI) solution at different irradiation time intervals after adding DPC.

Cr(VI) is a commonly found heavy metal pollutant with characteristics such as strong oxidizing ability, high toxicity, and carcinogenicity, posing significant risks to both environmental ecosystems and human health. Photocatalytic reduction is an effective method to convert toxic Cr(VI) into less hazardous Cr(III), thereby reducing its impact on the environment. The results of the photocatalytic experiments indicate that the reduction rate of Cr(VI) reaches approximately 96.04% within 30 min under light irradiation in the presence of ethanol and 10 mg of compound **1** (as the photocatalyst) in the solution (Figure 10a). 1,5-diphenylcarbazide (DPC) was employed as a chromogenic agent to indicate the concentration of Cr(VI). Under acidic conditions, Cr(VI) reacts with DPC to form a purple product, the intensity of which decreases as the Cr(VI) concentration diminishes. In contrast, Cr(III) does not react with DPC to produce any coloration [47]. As shown in Figure 10b, the purple color gradually faded with prolonged irradiation and became nearly colorless after 30 min, indicating that the concentration of Cr(VI) decreased significantly. This result is consistent with UV-Vis spectroscopy, confirming that compound **1** is an effective photocatalyst for Cr(VI) reduction.

To investigate the factors influencing the photocatalytic reduction of Cr(VI) by compound **1**, comparative experiments were performed (Figure S9). When the same amount of photocatalyst was used, the concentration of Cr(VI) remained almost unchanged both in the presence of ethanol without any light exposure and under light exposure without ethanol. Furthermore, the concentration of Cr(VI) remained virtually unchanged when ethanol and light exposure were both introduced but compound **1** was absent. The results demonstrate that compound **1** exhibits photocatalytic reduction activity toward Cr(VI), where ethanol likely acts as a h^+^ scavenger in the reaction process [48].

**Figure 11:**
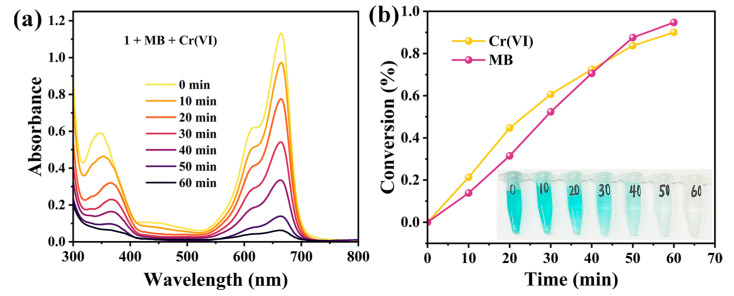
(**a**) Changes in UV-vis absorption spectra of a 50 mL mixed solution containing 50 ppm Cr(VI) and 40 ppm MB in the presence of **1**. (**b**) The corresponding photocatalytic degradation efficiency and the color changes in the mixed solution were observed at different irradiation time intervals.

Based on the above experimental results, we further conducted photocatalytic experiments using a mixed solution of Cr(VI) and MB. In total, 10 mg of compound **1** (serving as the photocatalyst) was dispersed in a 50 mL mixed solution (containing 50 ppm Cr(VI) and 40 ppm MB) for about 12 h under dark conditions to achieve adsorption-desorption equilibrium. Subsequently, 2.0 mL of ethanol was added in the mixture, and the mixture was then continuously stirred under illumination using a 300 W Xenon lamp with full spectrum, and the reaction timing was initiated. After 1 h, UV-Vis spectroscopy analysis revealed degradation efficiencies of 90.07% for Cr(VI) and 94.25% for MB (Figure 11). The results demonstrate that compound **1** can simultaneously degrade both Cr(VI) and MB in a mixed system.

In addition, we compared the photocatalytic degradation efficiency of compound **1** with other POMOCs, and the relevant data are detailed in Table S3. As can be seen from the table, the degradation efficiency of compound **1** for MB and Cr(VI) is comparable to the photocatalytic degradation capabilities of other POMOCs.

## 3. Experimental Section

### 3.1. Materials and Methods

All chemicals used were purchased directly from commercial sources without further purification. The IR spectra were recorded at room temperature in the range of 4000–500 cm^−1^ using a Perkin-Elmer Spectrum One FT-IR spectrometer (Waltham, MA, USA). Thermogravimetric (TG) analyses were performed on a Perkin-Elmer TGA7 instrument at a heating rate of 10 °C·min^−1^ from room temperature to 700 °C in the air atmosphere (Massachusetts, USA). Elemental analyses of C, H, and N were conducted using a Perkin-Elmer 2400 elemental analyzer (MA, USA). Powder X-ray diffraction (PXRD) patterns were carried out by a Bruker D8 Advance X-ray diffractometer (Billerica, MA, USA) using graphite monochromatized Cu-Ka radiation (λ = 1.5418 Å) in the 2θ range of 5–50° with an increment of 0.02 (Karlsruhe, Germany). All electrochemical tests were performed with a CHI760E workstation (Shanghai, China). UV-Vis absorption spectra were measured on a TU-1950 spectrophotometer (Beijing, China). Photocatalytic properties were tested using a CEL-HXF300 AULIGHT (Beijing, China).

### 3.2. Syntheses

#### Synthesis of [Ni(H_2_L)(HL)_2_](PMo_12_O_40_)·3H_3_O·4H_2_O (**1**)

A mixture of H_3_PMo_12_O_40_·H_2_O (0.091 g, 0.05 mmol), Ni(CH_3_COOH)_2_·4H_2_O (0.025 g, 0.1 mmol), H_2_L (0.019 g, 0.05 mmol), H_2_C_2_O_4_ (0.006 g, 0.05 mmol), and H_2_O (10 mL) was stirred for 30 min. The pH was adjusted to 2.0~2.5 with 1.0 M NaOH. Then, the mixture was transferred into a Teflon reactor and heated at 170 °C for 4 days, and finally cooled naturally to room temperature. Brown block crystals were obtained. Yield: 30% (based on Ni). Anal. Calc. for [Ni(H_2_L)(HL)_2_](PMo_12_O_40_)·3H_3_O·4H_2_O (C_60_H_57_N_12_O_59_Mo_12_NiP, 3131.13): C, 23.02; H, 1.83; N, 5.37 (%); Found: C, 23.15; H, 1.81; N, 5.84 (%). IR (KBr, cm^−1^): 3443 s, 3144 m, 1709 m, 1610 w, 1512 w, 1422 m, 1273 w, 1173 w, 1058 s, 960 s, 884 s, 804 s.

### 3.3. X-Ray Crystallography

Crystal data for **1** was collected on a Bruker APEX CCD area-detector at 293, with Mo Kα radiation for **1** (*λ* = 0.71076 Å, graphite monochromator). The structure was solved by direct methods and refined on *F*^2^ with full-matrix least-squares methods using the SHELXTL program. All non-hydrogen atoms were refined with anisotropic thermal parameters. The hydrogen atoms of the organic ligands and water molecules were placed theoretically. The CCDC reference number is 2385762. The crystallographic data and structural determinations were provided in Table 1, selected bond lengths and bond angles were given in Table S1, and hydrogen bond interactions were given in Table S2.

### 3.4. Preparation of Carbon Paste Electrode

The carbon paste electrode of **1** (**1**-CPE) was prepared as follows: graphite powder (0.5 g) and compound **1** (0.05 g) were accurately weighed and ground in a mortar for 30 min. Subsequently, paraffin oil (0.2 mL) was added, and the mixture was ground for an additional 30 min to obtain a uniformly mixed carbon paste electrode. Then, the mixture was compacted into a glass tube with an inner diameter of 3 mm. Finally, the copper rod as the electrical contact was inserted into the electrode from the back, and the surface of the electrode was polished until smooth with a weighing paper. The traditional three-electrode system was used: working electrode (**1**-CPE), auxiliary electrode (platinum wire), and reference electrode (Ag/AgCl).

### 3.5. Procedures of Photocatalysis

The procedure of the photocatalytic degradation of the organic dyes: First, 5.0 mg of compound **1** (as the photocatalyst) was dispersed in a 50 mL MB solution (40 ppm) for about 12 h under dark conditions (overnight) to reach adsorption–desorption equilibrium. The mixture was then continuously stirred under illumination from a 300 W xenon lamp with full spectrum. Then, 1.0 mL of sample was taken at a certain time interval for UV-Vis spectroscopy measurement.

The procedure of the photocatalytic reduction of Cr(VI): An aqueous Cr(VI) solution was prepared using K_2_Cr_2_O_7_ as the chromium source. Then, 10 mg of compound **1** (as the photocatalyst) was added to a 50 mL Cr(VI) solution (100 ppm), followed by the addition of 2.0 mL of ethanol. The mixture was then continuously stirred under illumination using a 300 W Xenon lamp with full spectrum, and the reaction timing was initiated. At specific time intervals, 1.0 mL of the sample was taken for UV-Vis spectroscopic measurement. For the colorimetric assay, each 1.0 mL sample was mixed with 9.0 mL of 0.2 M sulfuric acid solution, and 0.2 mL of a freshly prepared 1,5-diphenylcarbazide (DPC) solution (0.25% (*w*/*v*), dissolved in ethanol) was then added to the mixture, in which DPC is the chromogenic agent of Cr(VI). After thorough mixing, the solution was allowed to stand for 10–15 min. The change in Cr(VI) concentration was verified by observing the depth of the purple color of the mixed solution and compared with the results measured from UV-Vis spectroscopic analysis.

## 4. Conclusions

In this study, a 3D supramolecular structure of POMOC based on PMo_12_ was successfully synthesized by the hydrothermal method using the heterocyclic aromatic carboxylic acid ligand H_2_L. HS calculations revealed that hydrogen bonding is the predominant intermolecular force. The investigation of the electrochemical properties indicated that **1**-CPE exhibited bifunctional electrocatalytic properties for the reduction of NO_2_^−^, BrO_3_^−^, Cr(VI), and Fe(III), as well as for the oxidation of AA. In addition, **1**-CPE can be used as an efficient electrochemical sensor for detecting BrO_3_^−^ and Cr(VI), with the detection limits of 2.35 μM for BrO_3_^−^ and 1.17 μM for Cr(VI), respectively. Photocatalytic degradation studies revealed that compound **1** exhibited good photodegradation activity toward MB, achieving a photocatalytic degradation rate of 94.25% within 1 h using 5 mg of compound **1**. Radical trapping experiments indicated that ·OH served as the primary active species for dye decomposition. Tests of the photocatalytic reduction of Cr(VI) demonstrated a high photocatalytic reduction efficiency of 96.04% for a 100 ppm Cr(VI) solution within 30 min using 10 mg of **1**. Moreover, compound **1** exhibited a pronounced photocatalytic effect on the mixed solution of MB and Cr(VI). The experimental results showed that compound **1** is expected to be a multifunctional material for detecting and removing water pollutants.

## Figures and Tables

**Table 1:** Crystal data and structure refinements for compound **1**.

	1
Formula	C_60_H_57_N_12_O_59_Mo_12_NiP
Mol. wt.	3131.13
Crystal system	Monoclinic
Space group	C2/c
a (Å)	18.685 (2)
b (Å)	22.837 (2)
c (Å)	21.457 (2)
α (°)	90
β (°)	94.624 (5)
γ (°)	90
V (Å^3^)	9126.0 (16)
Z	4
Dc (g cm^−3^)	2.279
µ (mm^−1^)	1.922
F (000)	6080.9
Reflection collected	93,335
R_int_	0.1566
GOF	1.029
Final R ^ab^ indices [I > 2σ(I)]	R_1_ = 0.0505; wR_2_ = 0.0986
R indices (all data)	R_1_ = 0.0891; wR_2_ = 0.1131

^a^ R_1_ = ΣǀǀFoǀ − ǀFcǀǀ/ΣǀFǀ; ^b^ wR_2_ = ǀΣw (ǀFoǀ^2^ − ǀFcǀ^2^)^2^/Σǀw(F)^2^ǀ^1/2^.

## Data Availability

Data are contained within the article and Supplementary Materials.

## Supplementary Materials

The following supporting information can be downloaded at: https://www.mdpi.com/article/10.3390/molecules30102172/s1, Figure S1. Hydrogen bonds formed between carboxyl groups and interstitial crystal water molecules. Figure S2. The simulated and the experimental PXRD patterns of compound **1**. Figure S3. IR spectra of compound **1**. Figure S4. The TG and DSC curves of compound **1**. Figure S5. Band gap calculation for compound **1** (Inset: solid-state UV-Vis spectra). Figure S6. Changes in UV-Vis absorption spectra of MB solution in the presence of **1**. Figure S7. The photocatalytic cycle experiments of compound **1** on MB. Figure S8. The UV spectra of the Cr(VI) solution without adding catalyst (a), without C_2_H_5_OH (b), without 300W xenon lamp irradiation (c). Figure S9. The UV spectra of the Cr(VI) solution without adding catalyst (a), without C_2_H_5_OH (b), without 300 W xenon lamp irradiation (c); Table S1: Selected bond lengths (Å) and bond angles (°) for compound **1**. Table S2: Intermolecular hydrogen bonding for compound **1**; Table S3: Comparison of degradation rates of different POMOCs as photocatalysts.
