# Supplementary material for: A New [PMo12O40]3−-Based NiII Compound: Electrochemical and Photocatalytic Properties for Water Pollutant Removal
Source: Molecules. 2025 May 15;30(10):2172. doi: 10.3390/molecules30102172 (PMC12114273; doi:10.3390/molecules30102172)
Supplement: Supplementary file 1 [file molecules-30-02172-s001.zip › molecules-3638947-supplementary.pdf]

Supplement files

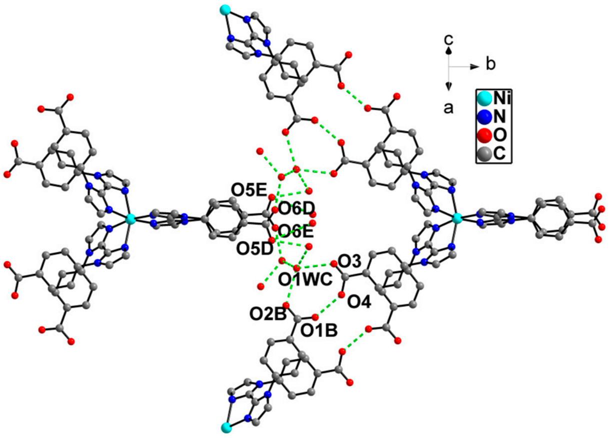

Figure S1. Hydrogen bonds formed between carboxyl groups and interstitial crystal water molecules.

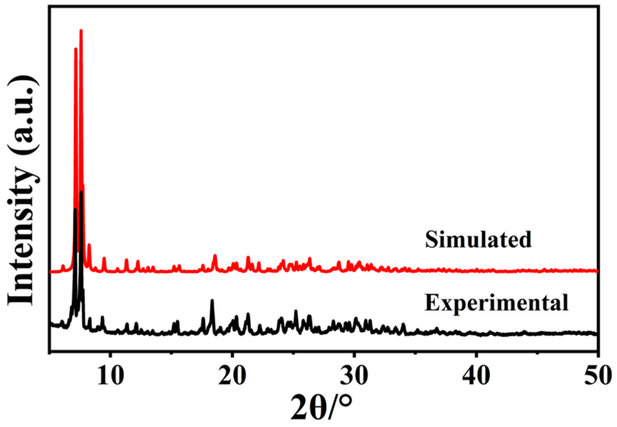

Figure S2. The simulated and the experimental PXRD patterns of compound **1**.

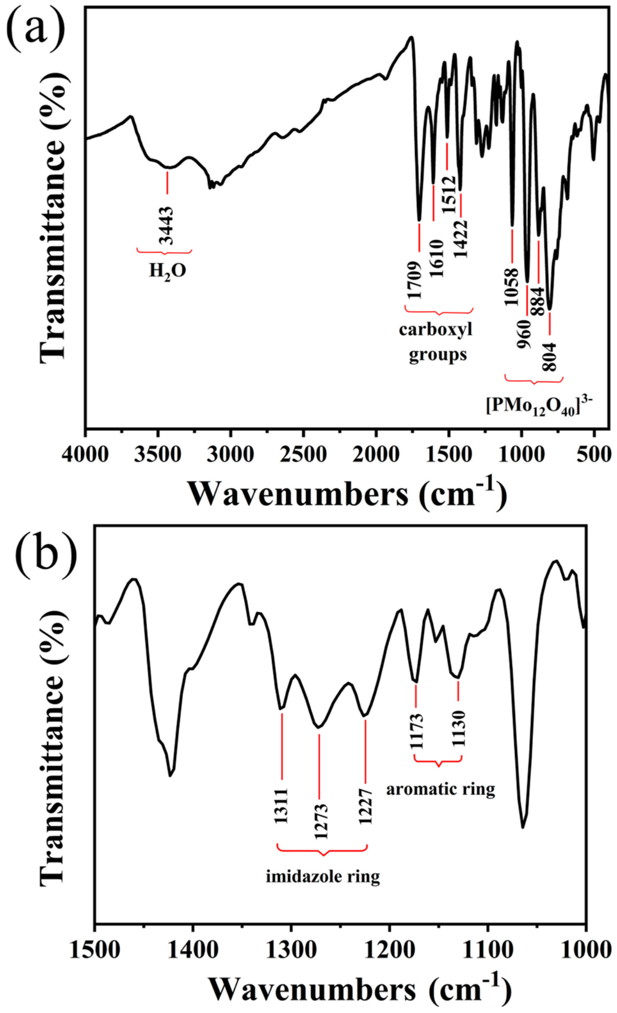

Figure S3. IR spectra of compound **1**.

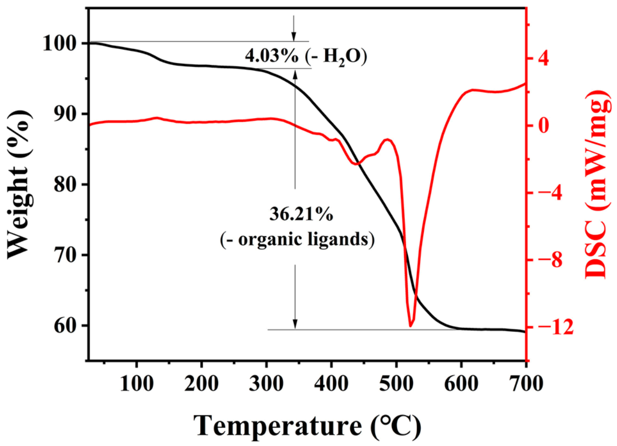

Figure S4. The TG and DSC curves of compound **1**.

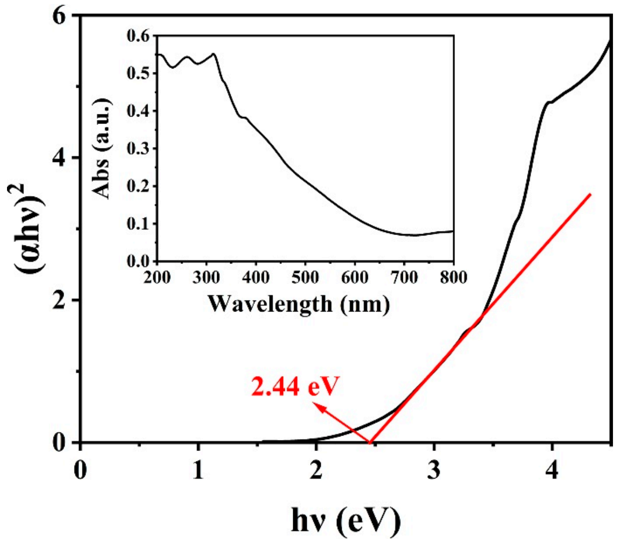

Figure S5. Band gap calculation for compound **1** (Inset: solid-state UV-Vis spectra).

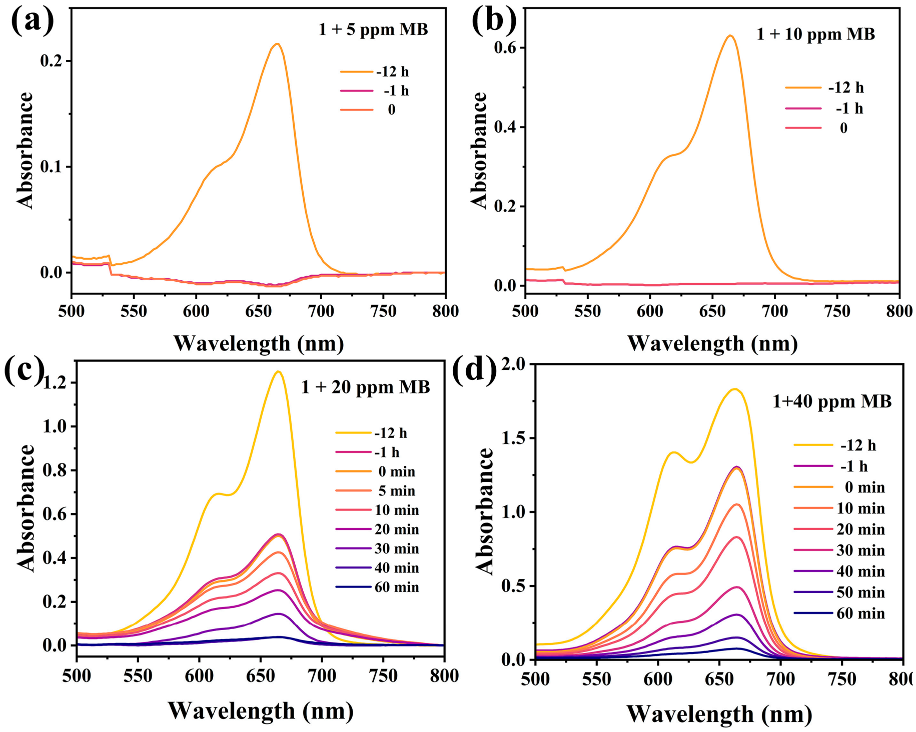

Figure S6. Changes in UV-vis absorption spectra of 5 ppm (a), 10 ppm (b), 20 ppm (c), and 40 ppm (d) MB solution in the presence of **1**.

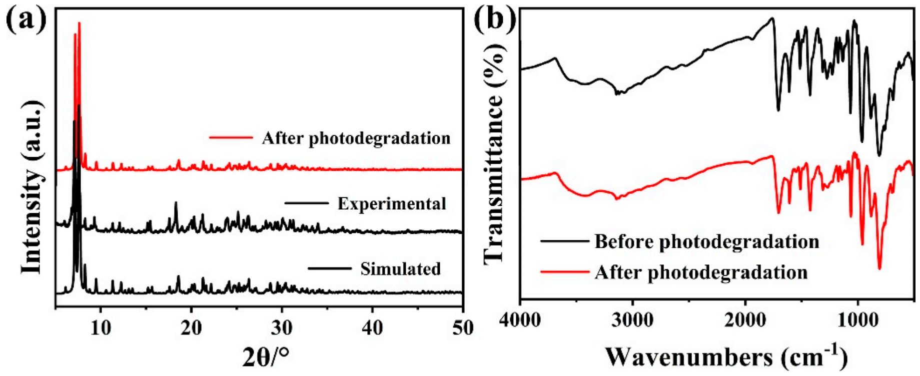

Figure S7. The PXRD (a) and FTIR (b) spectra of compound **1** remained unchanged before and after photocatalysis.

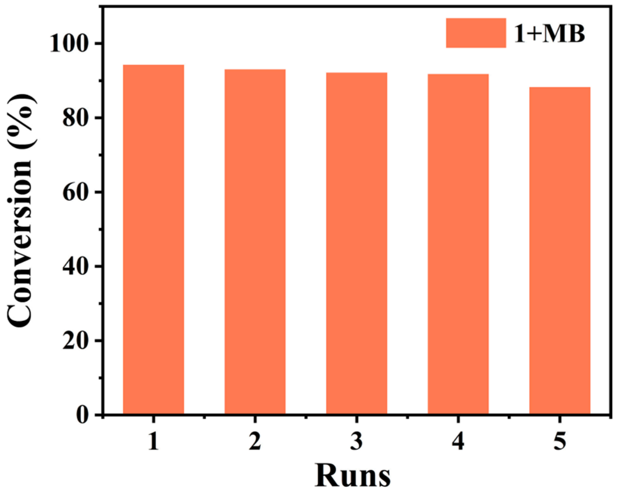

Figure S8. The photocatalytic cycle experiments of compound **1** on MB.

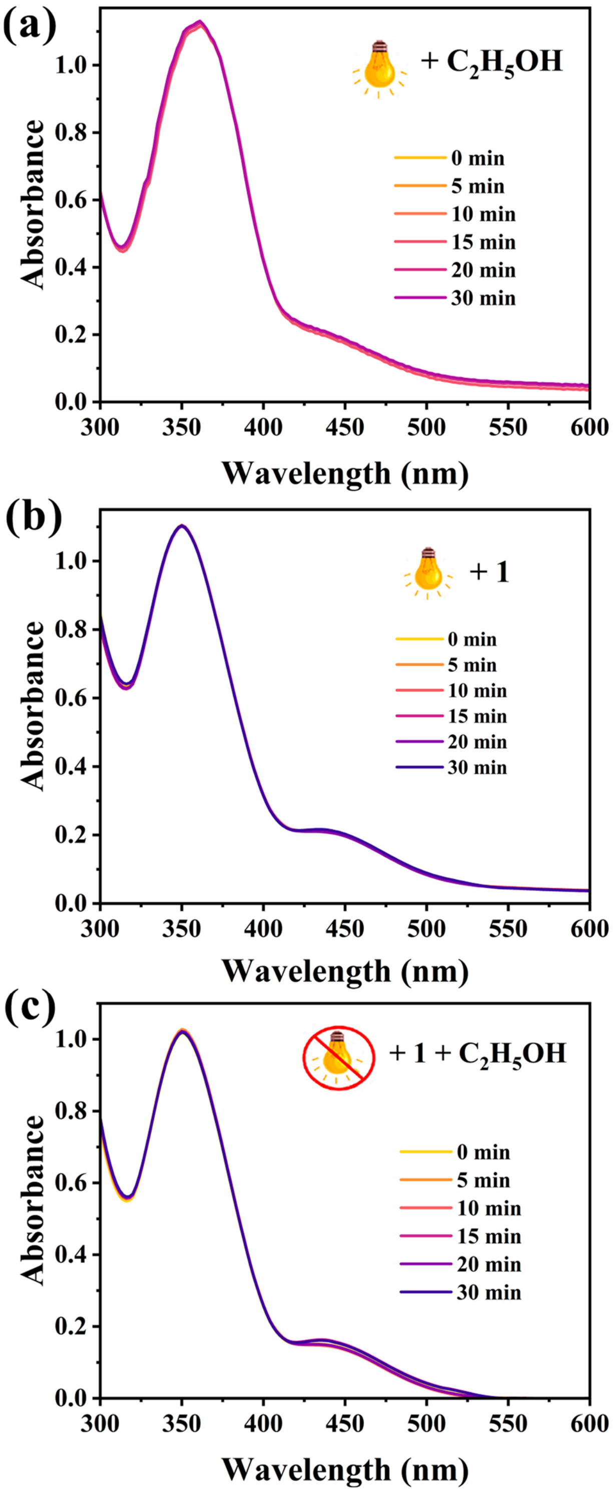

Figure S9. The UV spectra of the Cr(VI) solution without adding catalyst (a), without C_2H_5OH (b), without 300W xenon lamp irradiation (c).

Table S1 Selected bond lengths (Å) and bond angles (°) for compound **1**.

Mo5-O10	1.655(6)	P1-O24	1.506(10)
Mo5-O12	1.995(6)	Mo4-O14	1.833(7)
Mo5-O11	1.827(7)	Mo4-O25[1]	2.439(9)
Mo5-O8	1.985(7)	Mo4-O24[1]	2.487(10)
Mo5-O9	1.822(6)	Mo1-O11[1]	1.968(6)
Mo5-O26	2.436(9)	Mo1-O22	1.803(7)
Mo5-O24[1]	2.502(9)	Mo1-O20	2.016(7)
Mo3-O17	1.654(5)	Mo1-O21	1.649(6)
Mo3-O15	1.812(6)	Mo1-O23	1.827(6)
Mo3-O16	1.825(7)	Mo1-O27	2.465(9)
Mo3-O9[1]	1.981(6)	Mo1-O24	2.482(10)
Mo3-O18	1.973(7)	Mo2-O28	1.984(6)
Mo3-O26[1]	2.472(9)	Mo2-O20	1.804(7)
Mo3-O25[1]	2.482(10)	Mo2-O18	1.823(6)
Mo6-O7	1.649(6)	Mo2-O19	1.660(6)
Mo6-O16[1]	1.955(6)	Mo2-O14	1.972(7)
Mo6-O28	1.822(6)	Mo2-O25[1]	2.482(10)
Mo6-O8	1.820(7)	Mo2-O27	2.483(9)
Mo6-O23	1.970(7)	Ni1-N2	2.116(6)
Mo6-O26	2.457(10)	Ni1-N1	2.183(6)
Mo6-O27	2.482(10)	Ni1-N5	2.045(6)
Mo4-O12	1.825(6)	P1-O26	1.557(9)
Mo4-O13	1.647(6)	P1-O25	1.536(9)
Mo4-O15	1.980(6)	P1-O27	1.529(9)
Mo4-O22[1]	1.991(7)		

O10-Mo5-O12	101.2(3)	O22^{1}-Mo4-O25^{1}	90.4(3)
O10-Mo5-O11	102.9(3)	O22^{1}-Mo4-O24^{1}	62.9(3)
O10-Mo5-O8	101.6(3)	O14-Mo4-O15	87.4(3)
O10-Mo5-O9	101.6(3)	O14-Mo4-O22^{1}	155.4(4)
O10-Mo5-O26	158.6(3)	O14-Mo4-O25^{1}	65.0(4)
O10-Mo5-O24^{1}	159.4(3)	O14-Mo4-O24^{1}	96.0(4)
O12-Mo5-O26	90.4(3)	O25^{1}-Mo4-O24^{1}	41.3(3)
O12-Mo5-O24^{1}	63.5(3)	O11^{1}-Mo1-O20	79.4(3)
O11-Mo5-O12	86.8(3)	O11^{1}-Mo1-O27	89.4(3)
O11-Mo5-O8	153.9(3)	O11^{1}-Mo1-O24	63.4(3)
O11-Mo5-O26	95.6(3)	O22-Mo1-O11^{1}	87.7(3)
O11-Mo5-O24^{1}	64.5(3)	O22-Mo1-O20	154.5(3)
O8-Mo5-O12	79.7(3)	O22-Mo1-O23	96.3(3)
O8-Mo5-O26	62.5(3)	O22-Mo1-O27	95.6(3)
O8-Mo5-O24^{1}	89.5(3)	O22-Mo1-O24	65.1(3)
O9-Mo5-O12	156.0(3)	O20-Mo1-O27	62.6(3)
O9-Mo5-O11	95.6(3)	O20-Mo1-O24	89.4(3)
O9-Mo5-O8	88.2(3)	O21-Mo1-O11^{1}	100.8(3)
O9-Mo5-O26	65.6(3)	O21-Mo1-O22	104.0(4)
O9-Mo5-O24^{1}	96.0(4)	O21-Mo1-O20	100.0(4)
O26-Mo5-O24^{1}	41.4(3)	O21-Mo1-O23	103.4(4)
O17-Mo3-O15	101.5(3)	O21-Mo1-O27	158.1(4)
O17-Mo3-O16	101.5(3)	O21-Mo1-O24	160.0(4)
O17-Mo3-O9^{1}	101.8(3)	O23-Mo1-O11^{1}	153.6(3)
O17-Mo3-O18	102.4(3)	O23-Mo1-O20	86.3(3)
O17-Mo3-O26^{1}	157.6(3)	O23-Mo1-O27	64.3(3)

O17-Mo3-O25^{1}	159.2(3)	O23-Mo1-O24	94.7(4)
O15-Mo3-O16	95.2(3)	O27-Mo1-O24	40.7(3)
O15-Mo3-O9^{1}	155.7(3)	O28-Mo2-O25^{1}	89.5(3)
O15-Mo3-O18	87.1(3)	O28-Mo2-O27	63.5(3)
O15-Mo3-O26^{1}	96.3(3)	O20-Mo2-O28	87.9(3)
O15-Mo3-O25^{1}	63.6(3)	O20-Mo2-O18	96.4(3)
O16-Mo3-O9^{1}	86.7(3)	O20-Mo2-O14	155.2(4)
O16-Mo3-O18	155.0(3)	O20-Mo2-O25^{1}	96.5(4)
O16-Mo3-O26^{1}	63.1(3)	O20-Mo2-O27	64.5(3)
O16-Mo3-O25^{1}	94.6(4)	O18-Mo2-O28	155.3(3)
O9^{1}-Mo3-O26^{1}	63.0(3)	O18-Mo2-O14	87.2(3)
O9^{1}-Mo3-O25^{1}	92.0(3)	O18-Mo2-O25^{1}	65.8(3)
O18-Mo3-O9^{1}	81.4(3)	O18-Mo2-O27	96.4(4)
O18-Mo3-O26^{1}	91.9(3)	O19-Mo2-O28	99.7(3)
O18-Mo3-O25^{1}	64.1(3)	O19-Mo2-O20	102.7(4)
O26^{1}-Mo3-O25^{1}	43.2(3)	O19-Mo2-O18	103.0(4)
O7-Mo6-O16^{1}	99.9(3)	O19-Mo2-O14	100.4(4)
O7-Mo6-O28	103.7(4)	O19-Mo2-O25^{1}	158.9(4)
O7-Mo6-O8	103.7(4)	O19-Mo2-O27	158.0(4)
O7-Mo6-O23	99.3(3)	O14-Mo2-O28	79.3(3)
O7-Mo6-O26	157.3(3)	O14-Mo2-O25^{1}	62.5(3)
O7-Mo6-O27	157.9(3)	O14-Mo2-O27	90.7(3)
O16^{1}-Mo6-O23	80.9(3)	O25^{1}-Mo2-O27	41.9(3)
O16^{1}-Mo6-O26	62.0(3)	N2^{2}-Ni1-N2	89.7(3)
O16^{1}-Mo6-O27	89.6(3)	N2^{2}-Ni1-N1^{2}	79.6(2)
O28-Mo6-O16^{1}	155.0(4)	N2-Ni1-N1^{2}	85.5(2)

O28-Mo6-O23	87.3(3)	N2-Ni1-N1	79.6(2)
O28-Mo6-O26	96.6(3)	N1[2]-Ni1-N1	159.0(3)
O28-Mo6-O27	65.4(3)	N5-Ni1-N2[2]	96.9(2)
O8-Mo6-O16[1]	86.9(3)	N5-Ni1-N2	169.3(3)
O8-Mo6-O28	95.4(3)	N5-Ni1-N1[2]	103.9(2)
O8-Mo6-O23	155.5(4)	N5-Ni1-N1	92.5(2)
O8-Mo6-O26	63.9(3)	N5-Ni1-N5[2]	77.9(3)
O8-Mo6-O27	96.6(3)	O26-P1-O26[1]	180.0
O23-Mo6-O26	91.6(3)	O25-P1-O26[1]	107.7(5)
O23-Mo6-O27	62.4(3)	O25-P1-O26	72.3(5)
O26-Mo6-O27	43.1(3)	O25-P1-O25[1]	180.0
O12-Mo4-O15	154.9(3)	O27-P1-O26[1]	107.9(5)
O12-Mo4-O22[1]	86.5(3)	O27-P1-O26	72.1(5)
O12-Mo4-O14	96.4(3)	O27-P1-O25[1]	70.8(5)
O12-Mo4-O25[1]	96.4(3)	O27-P1-O25	109.2(5)
O12-Mo4-O24[1]	65.7(3)	O27[1]-P1-O27	180.0
O13-Mo4-O12	103.3(3)	O24-P1-O26[1]	69.5(5)
O13-Mo4-O15	100.0(3)	O24-P1-O26	110.5(5)
O13-Mo4-O22[1]	100.6(3)	O24[1]-P1-O25	110.4(5)
O13-Mo4-O14	102.4(4)	O24[1]-P1-O25[1]	69.6(5)
O13-Mo4-O25[1]	157.8(3)	O24-P1-O25	69.6(5)
O13-Mo4-O24[1]	159.6(3)	O24-P1-O27	69.0(5)
O15-Mo4-O22[1]	80.2(3)	O24-P1-O27[1]	111.0(5)
O15-Mo4-O25[1]	62.8(3)	O24-P1-O24[1]	180.0
O15-Mo4-O24[1]	89.3(3)		

[1] 3/2-*x*, 3/2-*y*, -*z*; [2] 1-*x*, +*y*, 1/2-*z*

Table S2 Intermolecular hydrogen bonding for compound **1**.

Donor-H···◎ cceptor	[◎ RU]	D - H	H···◎	D···◎	D- H···◎
O1W-H1W◎ ···O3	[6655.01]	0.85	2.26	2.762(12)	118
O1-H1◎ ···O4	[7655.01]	0.85	1.65	2.488(14)	168
O1W-H1WB···O2	[4565.01]	0.85	2.08	2.720(15)	132
O1W-H1WC···O2W	[1555.04]	0.85	1.9	2.49(4)	125
O3W-H3W◎ ···O3W	[2755.05]	0.85	2.45	2.781(17)	104
O3W-H3WB···O14	[1555.02]	0.85	2.55	3.185(12)	132
O5-H5◎ ···O2W	[6655.04]	0.85	1.79	2.64(3)	171
O5-H5◎ ···O3W	[6655.05]	0.85	2.37	3.102(13)	145
O4W-H4W◎ ···O6	[6645.01]	0.85	1.97	2.46(2)	115
O4W-H4WB···O1W	[2755.03]	0.85	1.66	2.44(2)	151
O4W-H4WC···O19	[1555.02]	0.85	1.64	2.390(19)	146
O5W-H5W◎ ···O4W	[1555.06]	0.85	1.52	2.36(3)	170
O5W-H5WB···O4W	[3765.06]	0.85	2.22	3.00(3)	153
O2W-H2W◎ ···O8	[1555.02]	0.85	2.57	2.95(4)	109
O2W-H2W◎ ···O5	[6645.01]	0.85	2.23	2.64(3)	110
O2W-H2WB···O3	[6655.01]	0.85	1.99	2.31(3)	102
C13-H13···O16	[7665.02]	0.93	2.25	3.095(11)	151

[7545] = [7_545] = 1/2-*x*, -1/2-*y*, -*z*; [5545.] = [3_545] = 1/2+*x*, -1/2+*y*, *z*; [4555] = [6_556] = *x*, -*y*, 1/2+*z*

Table S3 Comparison of degradation rates of different POMOCs as photocatalysts.

Catalyst	Substrates	Ccatalyst ($g{\cdot}L^{-1}$)	Time (min)	Degradation Rate (%)	Ref.
Compound 1	MB	0.10	60	94.25%	This work
	Cr	0.20	30	96.04%	This work
[◎ $g_4(H_2O)(L)_3(SiW_{12}O_{40})$] (L = 1,4-bis(3-(2-pyridyl)pyrazol)butane)	MB	0.50	160	83%	18
	Cr	0.50	160	100%	18
$CO(HL)_2(\beta\text{-}Mo_8O_{26})$	MB	0.63	210	20.7	38
$Ni(HL)_2(\beta\text{-}Mo_8O_{26})$	MB	0.63	210	13.9	38
$Zn(HL)_2(\beta\text{-}Mo_8O_{26})$	MB	0.63	210	81.6	38
$Cu_2(HL)_2(\mu_2\text{-}OH)_2(\beta\text{-}Mo_8O_{26})$	MB	0.63	210	49.4	38
$[Ni(mimb)_2(H_2O)_4(\beta\text{-}Mo_8O_{26})]\cdot 4H_2O$	Cr	0.01	20	100%	47
$[Zn\ (mimb)_2(H_2O)_4(\beta\text{-}Mo_8O_{26})]\cdot 4H_2O$	Cr	0.01	30	100%	47
